# Diagnostic performance of a faecal immunochemical test for patients with low-risk symptoms of colorectal cancer in primary care: an evaluation in the South West of England

**DOI:** 10.1038/s41416-020-01221-9

**Published:** 2021-01-19

**Authors:** Sarah E. R. Bailey, Gary A. Abel, Alex Atkins, Rachel Byford, Sarah-Jane Davies, Joe Mays, Timothy J. McDonald, Jon Miller, Catherine Neck, John Renninson, Paul Thomas, Fiona M. Walter, Sarah Warren, Willie Hamilton

**Affiliations:** 1 University of Exeter Medical School, St Luke’s Campus, Magdalen Road, Exeter, EX1 2LU UK; 2 Cancer Performance and Development Team, Royal Devon & Exeter NHS Foundation Trust, Barrack Road, Exeter, EX2 5DW UK; 3 Cancer Research UK, 2 Redman Place, London, E20 1JQ UK; 4 Peninsula Cancer Alliance, South West Clinical Networks & Senate, NHS England and NHS Improvement, South West House, Taunton, Somerset, TA1 2PX UK; 5 The Academic Department of Blood Sciences, Exeter Clinical Laboratory, Royal Devon & Exeter NHS Foundation Trust, Barrack Road, Exeter, EX2 5DW UK; 6 NHS South, Central, and West Commissioning Support Unit, South Plaza, Marlborough Street, Bristol, BS1 3NX UK; 7 Department of Clinical Biochemistry, Pathology Sciences Laboratory, Southmead Hospital, Westbury on Trym, Bristol, BS10 5NB UK; 8 The Primary Care Unit, Department of Public Health and Primary Care, University of Cambridge, Cambridge, CB1 8RN UK

**Keywords:** Digestive signs and symptoms, Diagnostic markers, Gastrointestinal cancer

## Abstract

**Background:**

The faecal immunochemical test (FIT) was introduced to triage patients with low-risk symptoms of possible colorectal cancer in English primary care in 2017, underpinned by little primary care evidence.

**Methods:**

All healthcare providers in the South West of England (population 4 million) participated in this evaluation. 3890 patients aged ≥50 years presenting in primary care with low-risk symptoms of colorectal cancer had a FIT from 01/06/2018 to 31/12/2018. A threshold of 10 μg Hb/g faeces defined a positive test.

**Results:**

Six hundred and eighteen (15.9%) patients tested positive; 458 (74.1%) had an urgent referral to specialist lower gastrointestinal (GI) services within three months. Forty-three were diagnosed with colorectal cancer within 12 months. 3272 tested negative; 324 (9.9%) had an urgent referral within three months. Eight were diagnosed with colorectal cancer within 12 months. Positive predictive value was 7.0% (95% CI 5.1–9.3%). Negative predictive value was 99.8% (CI 99.5–99.9%). Sensitivity was 84.3% (CI 71.4–93.0%), specificity 85.0% (CI 83.8–86.1%). The area under the ROC curve was 0.92 (CI 0.86–0.96). A threshold of 37 μg Hb/g faeces would identify patients with an individual 3% risk of cancer.

**Conclusions:**

FIT performs exceptionally well to triage patients with low-risk symptoms of colorectal cancer in primary care; a higher threshold may be appropriate in the wake of the COVID-19 crisis.

## Background

There are around 1.8 million new colorectal cancer (CRC) diagnoses worldwide each year, and almost 900,000 deaths.^1^ Population screening is effective in reducing mortality, with a relative risk of CRC mortality varying between 0.67 and 0.88 depending upon the screening modality, frequency of screening and sex.^2^ However, even when screening is available, most CRCs present with symptoms. In the UK, less than 10% of CRCs are identified by screening, with the remainder identified after symptoms have developed.^3^ In many countries, symptomatic patients present first to primary care, where the general practitioner (GP) assesses the possibility of cancer, and investigates or refers for specialist tests if appropriate.^4^ The usual diagnostic test in secondary care is colonoscopy, with CT imaging or capsule endoscopy occasionally used.

Requests for urgent CRC investigation have relentlessly increased over the last decade, with a parallel increase in colonoscopies. These doubled in the UK between 2012 and 2017.^5^ This rise was driven in part by referral of patients whose symptom profile, while still representing possible cancer, was relatively low-risk.^6^ These patients, often with abdominal pain or mild anaemia, had been excluded from UK national guidance in 2005,^7^ but transpired to have the worst survival across the different symptoms, often presenting as an emergency.^8,9^ In 2015, the National Institute for Health and Care Excellence (NICE) published revised guidance, NG12.^10^ The revised NICE recommendations were explicitly based on the risk of cancer posed by the patient’s symptoms, and used only primary care evidence to estimate this risk. Patients having a risk of CRC of 3% or more are recommended for an urgent suspected cancer referral, and are usually offered colonoscopy. For risks below 3%, patients were to be offered testing for occult blood in their faeces, with those testing positive to be referred urgently. This recommendation was based on a systematic review performed by NICE, finding six studies of faecal occult blood testing mostly in secondary care, totalling 9871 patients.^10^ The sensitivity and specificity for colorectal cancer varied considerably across these studies, although the diagnostic performance was considered sufficient in the absence of other tests available in primary care. An economic evaluation supported this recommendation.^10^

The faecal immunochemical test (FIT) for haemoglobin measures the amount of haemoglobin in a faeces sample and has largely replaced faecal occult blood testing. NICE guidance issued in 2017 (DG30) recommended FIT should replace faecal occult blood testing in primary care patients with low-risk symptoms of CRC.^11^ The systematic review underpinning that recommendation found nine studies:^12^ in only one was the FIT performed in primary care, though even in that study all patients had already been selected for urgent referral for possible CRC.^13^ Thus all the evidence underpinning the use of FITs in primary care in DG30 was from the high-risk referred population; this brings a substantial risk of spectrum bias.^14^

This study evaluated a FIT used by general practitioners to triage patients with low-risk symptoms of possible CRC in the South West of England, and estimated the diagnostic performance of FITs in this population.

## Methods

This joint South West Cancer Alliances transformation project provided a quantitative FIT service to primary care practices across the South West of England (population ~4 million) from June 2018. This area includes 14 secondary care providers and 10 clinical commissioning groups (CCGs), listed in Supplementary Material.

The FIT diagnostic service (comprising of FIT kits for patients, patient instructions, lab processing of FIT and timely associated reporting of results) was available to GPs to triage patients with low-risk symptoms of CRC, as defined by NG12 and DG30.^10,11^ Patients meeting the following criteria were eligible for a FIT (these criteria were derived from the 2015 NG12 for faecal occult blood testing, current at the time of project design):

- Aged 50 years and over with unexplained abdominal pain or weight loss
- Aged 50–60 years with change in bowel habit or iron-deficiency anaemia
- Aged 60 years and over with anaemia, even in the absence of iron deficiency

The laboratory service was provided by Severn Pathology in Bristol and the Exeter Clinical Laboratory in Exeter using the HM-JACKarc analyser. This assay has a recommended analytical range of 7–400 μg Hb/g faeces (though some values below 7 μg Hb/g faeces were reported); results over 400 μg Hb/g faeces were recorded as >400 μg Hb/g faeces. A threshold value of ≥10 μg Hb/g faeces defined a ‘positive’ test, as per DG30.^11^ Test kit packs were delivered to primary care practices including: the test unit, instructions for use, a form to select the indication for the test, and a prepaid envelope for the patient to return the completed test to the laboratory. Test results were returned to practices electronically in Devon, Cornwall and Avon. In Somerset, Wiltshire and Gloucestershire, reports were initially sent by post, and later electronically.

Information about the service was publicised through local CCG newsletters and through the local Cancer Research UK Facilitator Team, who provided practice-level training and support. GPs were provided with written, online, and video support for using the FIT service, indications for the test and how to use it, and advice on how to deal with a positive test. GPs were advised in the guidance that if faecal haemoglobin concentration (f-Hb) was ≥10 μg Hb/g faeces they should consider using an urgent referral for suspected cancer under the local secondary care provider’s arrangements. They were also advised that occult blood in the faeces can be caused by a wide variety of benign conditions as well as CRC, and further assessment may be appropriate to rule these out before referring.

### Data collection

All patients with a FIT analysed from 1 June 2018 to 31 December 2018 were included in this study. Data extracted from the two laboratories included the test date, result, indication, patient year of birth and gender. Separately, each of the 14 secondary care providers in the region extracted data, including stage at diagnosis, on any cancer identified from 1 June 2018 to 31 December 2019 after entry into upper or lower gastrointestinal services. This captured cancers diagnosed by all routes, including screening and incidental findings such as routine referral or emergency admission. This allowed for 12 months of follow-up time for all patients, during which missed CRC diagnoses in FIT negative patients were likely (but not certain) to be diagnosed through other routes. Had a longer period of follow-up been chosen, some of the CRCs diagnosed in FIT negative patients after 12 months may not have been causing symptoms at the time of testing. Only cancers identifiable on a gastrointestinal (GI) pathway were identified: non-GI cancers, referred to other cancer diagnostic pathways, were not identified.

Test results were matched against referral and diagnosis data by each of the secondary care providers using NHS number, then removed and replaced with a randomly allocated study number. Year of birth was used as a secondary confirmation of correct matching of patient records. This was done to adhere with information governance requirements and to ensure completeness of the full patient pathway. GPs were advised not to offer multiple FITs to individual patients; where more than one test was recorded for one patient, the earliest result was used.

### Statistical analysis and power calculation

Summary statistics were used to describe the cohort, and to estimate the performance of FIT in this population, including sensitivity, specificity, positive predictive value (PPV), and negative predictive value. A Chi-squared test was used to compare the proportion of male participants, and a Mann–Whitney test to compare the median age, between those with a result at/above and below the threshold. A receiver-operating characteristic curve was produced for quantitative f-Hb against CRC diagnosis. Logistic regression was used to model the relationship between cancer and f-Hb (treated as a continuous variable), after log-transformation to improve the final model fit. Non-linearity in the relationship between f-Hb and CRC was explored using fractional polynomials, though goodness of fit was not improved by doing so. Consequently, a linear term was retained. The probability of being diagnosed with CRC in the next year for a given f-Hb value was estimated from the final model, in particular identifying the value equating to an individual cancer risk of 3%, to mirror NICE recommendations for urgent investigation.^10^ Stata version 16 was used for all analyses.^15^ Diagnostic test summary statistics were estimated with the DIAGT module.^16^

A simulation approach was used to estimate the sample size required to achieve 95% confidence intervals of 2.2% to 4.0% around a cancer risk of 3% from the logistic regression. Assuming a linear relationship between f-Hb and CRC risk suggested a sample of 2250 would be sufficient so long as the threshold was within the central core of the distribution of f-Hb levels. It was estimated that 10,000 tests would be used in a year; data were collected over seven months to meet the sample size requirement. In practice it was comfortably exceeded, increasing precision.

### Data governance

As this project was evaluating service delivery, and not changing routine clinical practice, ethical approval was not required. Data sharing agreements were drawn up between all parties, and Caldicott guardian approvals were in place to allow data sharing. The requirement for individual NHS numbers for use within this evaluation meets the criteria set out in section 6 of the General Data Protection Regulation: Guidance on Lawful Processing. The processing of data is based upon GDPR Article 6(1)(e)—‘exercise of official authority’ and article 9(2)(h) ‘management of health and care services’. The enabling legislation is the NHS Act 2006 section 13E, including the duty on NHS England to ‘secure continuous improvement in the quality of services’. The same basis supported the secondary care providers supplying data. The study protocol is available on the University of Exeter website at http://hdl.handle.net/10871/122303. This manuscript was deposited as a preprint on medrXiv.^17^

## Results

From 1 June 2018 to 31 December 2018, 3890 samples were submitted to and analysed by the two laboratories. The median age of tested patients was 65 years (interquartile range (IQR): 56–75) and 1644 (42.6%) were male. Criteria for investigation were: 1617 (41.6%) aged ≥50 years with abdominal pain or weight loss; 1194 (30.7%) aged <60 years with changes in bowel habit or iron-deficiency anaemia; 930 (23.9%) aged >60 years and with anaemia (in absence of iron deficiency). No criteria for investigation were recorded in 149 (3.8%).

### FIT results

**Fig. 1:**
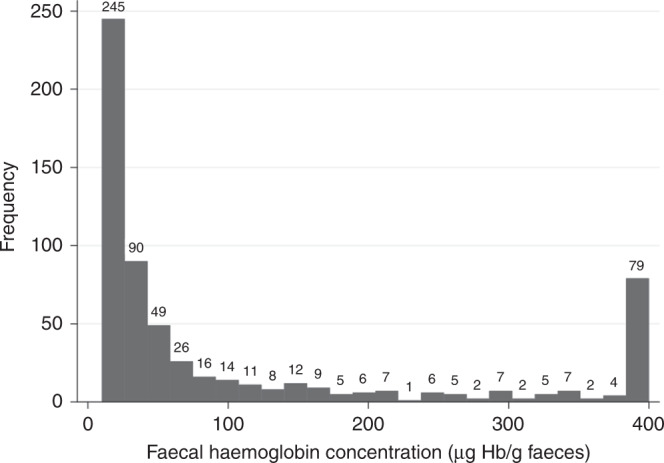
Histogram of quantitative faecal immunochemical test results. This histogram presents the quantitative faecal immunochemical test results for patients with a f-Hb over the threshold of 10 μg Hb/g faeces (618 primary care patients), with low-risk symptoms of possible colorectal cancer.

A f-Hb ≥10 μg Hb/g faeces (test positive) was recorded for 618 patients (15.9%). Patients above the f-Hb threshold were more often male (46.1% males vs 42.0% females, *p* = 0.017) and older (median age 71 vs 63, IQR 60 to 79, *p* < 0.001). Of patients above the f-Hb threshold, the median age was 71.7 years (IQR 60.1–79.7); 288 (46.3%) were male. The median result was 36 μg Hb/g faeces (IQR 17–149). Figure 1 shows the distribution of f-Hb in patients above the threshold.

### Referrals in patients with a FIT

**Fig. 2:**
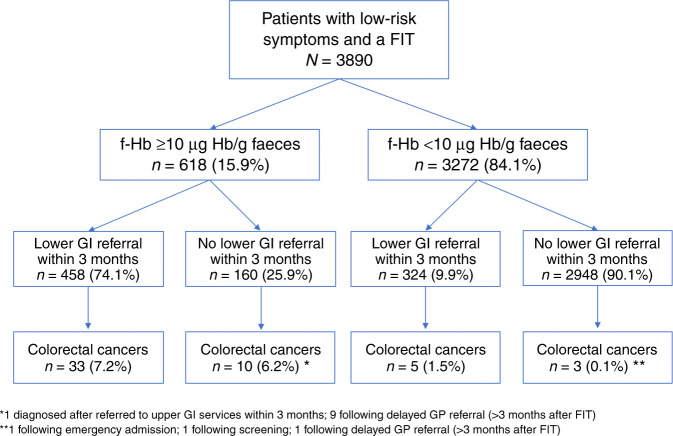
Flow diagram of referrals and diagnoses in patients with a FIT. This flow diagram shows the number of patients tested, split by test result (over/at or under the threshold), and subsequent lower gastrointestinal referrals and colorectal cancers diagnosed.

Of 618 patients with f-Hb ≥10 μg Hb/g faeces, 458 (74.1%) were referred to lower gastrointestinal (GI) services within three months (Fig. 2). Of the remaining 160, 36 were referred up to 12 months after FIT. Cancer outcomes for these patients are shown in Fig. 2. Of 3272 patients with f-Hb <10 μg Hb/g faeces, 324 (9.9%) were referred to lower GI services within three months.

### Cancer outcomes

**Fig. 3:**
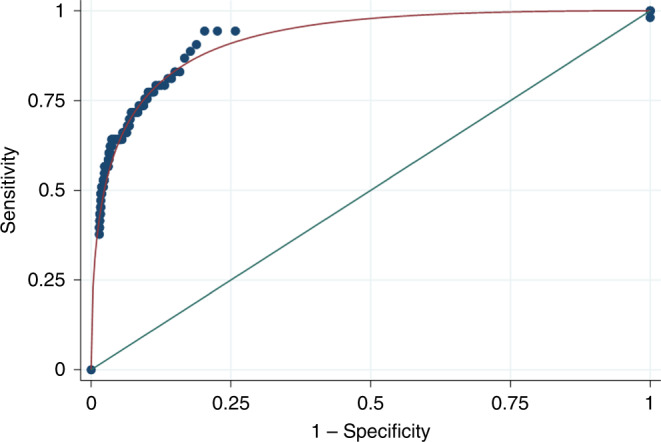
Receiver operator characteristic (ROC) curve for the faecal immunochemical test in the low-risk symptomatic primary care population. Area under the curve = 0.92 (CI 0.86–0.96).

Table 1 shows the cancers identified during the year after FIT. The positive predictive value of FIT in this low-risk symptomatic population is 7.0% (95% CI 5.1–9.3%), and the negative predictive value is 99.8% (CI 99.5–99.9%). The sensitivity in this population is 84.3% (CI 71.4–93.0%), and the specificity 85.0% (83.8–86.1%). The area under the ROC curve is 0.92 (CI 0.86–0.96) (Fig. 3).

**Table 1:** The number of colorectal, oesophago-gastric and pancreatic cancers diagnosed in total, for patients with f-Hb at/above and below the 10 μg Hb/g faeces threshold, within 12 months of FIT date.

Cancer	Total	≥10 μg Hb/g faeces	<10 μg Hb/g faeces
		*n* = 618	*n* = 3272
Colorectal	51	43 (7.0%)	8 (0.2%)
Oesophago-gastric	9	3 (0.5%)	6 (0.2%)
Pancreatic	7	1 (0.2%)	6 (0.2%)
Total	67	47 (7.6%)	20 (0.6%)

The median number of days from FIT to diagnosis of CRC in patients testing above the f-Hb threshold was 34 (IQR 23–56). Staging data were available for 31 of 43 patients: 6 Dukes’ A; 5 Duke’s B; 12 Dukes’ C; 8 Dukes’ D. The median number of days to diagnosis in patients with a result below the f-Hb threshold was 57 days (IQR 37–197). Staging data were available for six of eight patients: 1 Duke’s B; 2 Dukes’ C; 3 Dukes’ D.

### Cancer risk by f-Hb

**Fig. 4:**
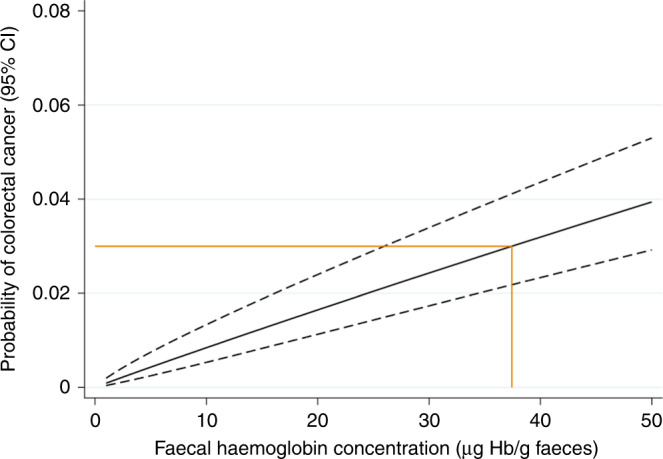
Predicted probability of colorectal cancer against f-Hb, with 95% confidence limits. The orange line shows the f-Hb level that corresponds to a colorectal cancer risk of 3% in an individual with that result.

Figure 4 shows the estimated probability that an individual will be diagnosed with CRC for a given f-Hb level, estimated from the logistic regression model. Using this model, a f-Hb level of 37 μg Hb/g faeces (CI 26–50) in an individual with that result corresponds to a CRC risk of 3%. Five patients with CRC had a f-Hb value in the range 5–9 μg Hb/g faeces.

## Discussion

This study reports the use of FIT for detection of CRC in a primary care symptomatic population. The test performed very well using the threshold value of ≥10 μg Hb/g faeces. Test sensitivity and specificity were 84.3% and 85.0%, respectively, both notably high figures for a primary care cancer test. Using this threshold, the positive predictive value of f-Hb ≥10 μg Hb/g faeces was 7.0%, and the negative predictive value 99.8%, in a population with an overall prevalence of CRC of 1.3%. FIT also performed well irrespective of gender or age. A f-Hb level of 37 μg Hb/g faeces corresponded to an individual’s CRC risk of 3%.

### Strengths and limitations

The strengths of this study are its size, and its setting being where the test will be used, eliminating spectrum bias. The three symptom groupings used by GPs to prompt FIT were estimated to have PPVs in primary care in the range 1–3%, and the overall prevalence of 1.3% fell within that range. These defined symptoms match the current (September 2020) NICE guidance on when to offer faecal testing for colorectal cancer to adults without rectal bleeding.^10^ Every one of the 14 secondary care providers in the region were recruited, increasing reliability and generalisability. Cancer metrics in the NHS are very accurately maintained; dedicated cancer managers ensure accurate data recording, and secondary care provider performance on cancer metrics is regularly published in the public domain. Furthermore, all secondary care providers of cancer services within England are required to use nationally defined datasets eliminating disparity in data definitions. Despite the thorough methods, it is possible that a small number of cancers were missed, although this is unlikely to affect the overall interpretation of the results. Crucially, the methods allowed the identification of CRCs in those not offered further investigation after the FIT result was received, and a long follow-up period of 1 year was achieved.

The age group studied, with a median age of 65 years for those tested, is close to the median age for CRC diagnosis of 72 years, suggesting the GPs were using the test in those genuinely considered to have a real—but small—risk of cancer. More women were tested, whereas CRC is slightly more common in men. This may reflect the entry criteria, particularly with two of the three criteria incorporating iron-deficiency anaemia, a condition more common in women,^18^ or the fact that women are more likely to seek medical intervention.^19^ Symptom data could not be verified, but the overall prevalence figure suggests testing was rarely extended into higher-risk groups. Completing the test was patient driven; only tests which were completed and returned to the lab were reported; it is not known how many tests were handed out by GPs and not returned to the labs. Both participating laboratories used the same FIT system; achieving consistency across the cohort, but meaning the results are not applicable to other systems.^20^

### Comparison with previous literature

Three recent studies can be compared, as they examined FIT in the symptomatic primary care population, rather than the screening or referred populations: two of these also used a threshold of 10 μg Hb/g faeces. Juul et al. studied 3462 Danish primary care patients aged ≥30 years, with symptoms not meriting urgent colonoscopy, but not defined further.^21^ In that study, FIT was also recommended in patients diagnosed with irritable bowel syndrome, lest this were a misdiagnosis. 15.6% patients tested over the threshold, and 9.4% of these (CI 7.0–11.9%) had a CRC diagnosed in the next 3 months. There were fewer than three cancers identified in those below the threshold (the inexact number reflecting Danish data protection rules). Nicholson et al. followed up 9896 primary care patients in Oxfordshire, England, for 6 months after FITs were ordered in primary care. The entry criteria did not match NICE guidance DG30 or NG12, and included rectal bleeding. The sensitivity for CRC was 90.5% (CI 84.9–96.1%), and the positive predictive value of a positive test 10.1% (CI 8.2–12.0%).^22^ Chapman et al*.* stratified patients presenting in primary care with any lower GI symptoms (except rectal bleeding or a rectal mass) by f-Hb level, and anaemia in one strata.^23^ PPVs were: 30.0% in the ≥150.0 μg Hb/g faeces group; 4.4% in the 10.0–149.9 μg Hb/g faeces group; 0 in the 4.0–9.9 μg Hb/g faeces with anaemia group, 2.9% in the 4.0–9.9 μg Hb/g faeces without anaemia group and 0.2% in the <4.0 μg Hb/g faeces group. The PPV of 7% for results over the 10 μg Hb/g faeces threshold in the present study is the lowest of the three that used that threshold, which may reflect the stricter criteria for use, in particular excluding patients with rectal bleeding, but matching current national guidance.^10^ The PPV in patients with f-Hb from 4.0–9.9 μg Hb/g faeces in our cohort was 1.7%; comparable to Chapman et al.*’s* results.^23^ As a comparison, the sensitivity of 84.3% reported here is higher than in the screening population of 67.0% (CI 59.0–74.0%, with thresholds of >50 μg/g^24^), and lower than that in referred populations of 93.3% (CI 80.7–98.3%, thresholds of >10 μg/g).^25^

### Clinical and research use of the results

The values reported in this study are excellent for a cancer triage test in primary care. The performance of diagnostic tests is generally worse as the prevalence of the target condition falls.^14^ In primary care, gastrointestinal complaints are common, and the symptoms of CRC overlap both with less common cancers, such as pancreatic or oesophago-gastric, and with common benign conditions. With most symptoms (apart from rectal bleeding) the likelihood of CRC is low, often in the range 1–3%.^26^ A large UK primary care study showed that patients would opt for cancer investigation even for risks as low as 1%.^27^ FIT has been introduced to allow primary care investigation of such patients. It works in classical Bayesian fashion: from a prior risk of CRC of 1.3% in the symptomatic population, a result over the 10 μg Hb/g faeces threshold increased the risk to 7.0%, and a result below the threshold reduced it to 0.2%, which is approximately the whole population background risk, including those without symptoms.^26^ Furthermore, in five of the eight cancers with a result under the threshold, the patient’s GP still requested urgent investigation for possible CRC, probably because continuing symptoms allowed the GP to ‘overrule’ the negative test. Conversely, nearly a quarter of patients who tested over the threshold were not offered investigation within three months, although ultimately all who had CRC were investigated within a year. CRC incidence was similar in those who were referred within three months and those who were referred later; delays in the referral process should be avoided.

Other cancers were diagnosed in participants in this study. Sixteen oesophago-gastric or pancreatic cancers were found, only four having a FIT result over the threshold. This suggests that GPs should consider other intra-abdominal cancers in patients with f-Hb < 10 μg Hb/g faeces and continuing symptoms, even though FIT is intended for the detection of colorectal cancers as Hb is immunologically degraded in the small intestine.

While the PPV at or above the current threshold of 10 μg Hb/g faeces is 7%, the risk of having CRC for an individual with f-Hb of exactly this value is 1% or lower (Fig. 4). Given this risk is lower than the 3% chosen to underpin the NICE NG12 recommendations for urgent cancer investigation,^10^ there may be scope to raise the threshold at which urgent definitive investigations are undertaken. f-Hb of 37 μg Hb/g faeces (CI 26–50) would identify those with a personal 3% risk of cancer, though the large uncertainty on this estimate may warrant the use of a lower value until more data are available to reduce this uncertainty. Such a change may be appropriate while endoscopy resources are severely curtailed by COVID-19 precautions, with ‘safety netting’ by GP review for those with f-Hb levels between 10 and 36 μg Hb/g faeces.^28^ In the long term, however, the UK’s aspiration is for improvements in cancer diagnosis to increase the proportion of cancer patients diagnosed at stage I or II to 75% by 2028 (from a pre-COVID ~53%).^29^ If CRC improvements are to contribute to this target, it may be that the threshold should be retained at 10 μg Hb/g faeces, or even lowered further, though not below the level where the test is considered reliable, currently 7 μg Hb/g faeces.

Several research needs arise from this study. The first is a health-economic analysis, examining the choice of f-Hb threshold from that perspective. Second, it may be possible to combine data on symptoms, other lab tests, and demographics with f-Hb to increase the predictive power of FIT. A third strand of research—not directly related to this study, but overlapping—considers whether FIT can be used to triage the high-risk population. Such studies are underway; in Scotland, patients reporting rectal bleeding (considered a ‘red flag’ symptom) in primary care with a f-Hb <10 μg Hb/g faeces were unlikely to be harbouring CRC or other serious bowel disease;^30^ similar results were observed in Sweden.^31^ Those will complement the study reported here, and establish the final place for FIT in colorectal cancer triage.

## Conclusion

FIT in the low-risk primary care population performs well. False-negatives are few in number, and many of those with a false-negative test appear to receive timely investigation despite f-Hb below the 10 μg Hb/g faeces threshold. The false-positive rate is 93%, meaning 13 patients with f-Hb over 10 μg Hb/g faeces have to undergo colonoscopy to identify one CRC. This is a major diagnostic advance; low-risk patients were previously either not investigated, and had more emergency admissions and worst survival,^8,9^ or were referred for colonoscopy. The background rate of cancer of 1.3% in this population meant 77 patients had to be offered colonoscopy to identify one cancer, potentially swamping endoscopy services, and putting patients at a small risk of complications. Clinically, therefore, FIT works, although health-economic aspects are as yet uncertain.

## Supplementary information

Supplementary material

## Data Availability

As the raw data analysed in this study include patient identifiable information, we cannot share them currently. We are happy to discuss requests for data with the Caldicott guardian (J.R.).
