# Supplementary material for: Diagnostic performance of a faecal immunochemical test for patients with low-risk symptoms of colorectal cancer in primary care: an evaluation in the South West of England
Source: Br J Cancer. 2021 Jan 19;124(7):1231–6. doi: 10.1038/s41416-020-01221-9 (PMC8007716; doi:10.1038/s41416-020-01221-9)
Supplement: Supplementary file 1 — Supplementary material [file 41416_2020_1221_MOESM1_ESM.docx]

Supplementary material

Trusts included in the study area:

- Gloucestershire Hospitals NHS Foundation Trust
- Great Western Hospitals NHS Foundation Trust
- North Bristol NHS Trust
- Northern Devon Healthcare NHS Trust
- Royal Cornwall Hospitals NHS Trust
- Royal Devon and Exeter NHS Foundation Trust
- Royal United Hospitals Bath
- Salisbury NHS Foundation Trust
- Taunton and Somerset NHS Foundation Trust
- Torbay and South Devon NHS Foundation Trust
- University Hospitals Bristol NHS Foundation Trust
- University Hospitals Plymouth NHS Trust
- Weston Area Health NHS Trust
- Yeovil District Hospital NHS Foundation Trust

Clinical commissioning groups (CCGs) in the study area:

- NHS Bath and North East Somerset, Swindon and Wiltshire CCG (this includes the merged NHS Bath and North East Somerset CCG, NHS Swindon CCG and NHS Wiltshire CCG)
- NHS Bristol, North Somerset and South Gloucestershire CCG
- NHS Devon CCG
- NHS Dorset CCG
- NHS Gloucestershire CCG
- NHS Kernow CCG
- NHS Somerset CCG
